# Shift work and metabolic dysfunction-associated steatotic liver disease: a systematic review of observational studies

**DOI:** 10.1007/s00420-025-02171-6

**Published:** 2025-09-17

**Authors:** Bingya Ma, Yihang Fan, Wenjun Fan

**Affiliations:** Department of Epidemiology and Biostatistics, University of California, Irvine, Irvine, CA USA

**Keywords:** Shift work, MASLD, MASH, Circadian disruption, Systematic review

## Abstract

**Objective:**

Shift work disrupts the circadian rhythm and may increase the risk of metabolic disorders, including nonalcoholic fatty liver disease, recently redefined as metabolic dysfunction-associated steatotic liver disease (MASLD), and its progressive form, metabolic dysfunction-associated steatohepatitis. This systematic review aimed to synthesize observational studies on the association between shift work and MASLD.

**Methods:**

A comprehensive literature search was conducted in the PubMed, Scopus, and Web of Science databases up to November 25, 2024, following the Preferred Reporting Items for Systematic Reviews and Meta-Analyses guidelines. Data were extracted and summarized based on pre-specified inclusion and exclusion criteria. The National Institutes of Health quality assessment tool was used to evaluate the quality of the included studies. Both data extraction and quality assessment were conducted independently by two authors, with disagreements resolved through consensus.

**Results:**

Nine studies met the criteria and were included in the review, including various occupational groups. Most studies reported a positive association between shift work and MASLD, with stronger effects observed in workers exposed to long-term or frequent shift work. Subgroup and interaction analyses suggested that gender, age, lifestyle, chronotype, and occupational factors may modify this association, while body mass index was identified as a potential mediator of the relationship between shift work and MASLD. However, methodological issues, such as imprecise exposure and outcome measurements and a lack of time-varying analysis, limit causal interpretation.

**Conclusion:**

The systematic review supports an association between shift work and increased MASLD risk. Further prospective studies with rigorous designs and diverse populations, as well as stronger mechanistic evidence, are needed to establish a causal link between shift work and MASLD.

**Supplementary Information:**

The online version contains supplementary material available at 10.1007/s00420-025-02171-6.

## Introduction

Shift work is defined as the alternate and rotating morning, afternoon, and night shifts, with employees frequently working outside the typical 7:00 AM–6:00 PM hours (Straif et al. 2007). Shift work is common in several sectors, including transportation, telecommunications, broadcasting, health care, and food production (Matheson et al. 2014). In the US, around 20% of the working population practiced this work pattern (Alterman et al. 2013). Since shift employment often involves night work, the typical sleep–wake cycle (circadian rhythm) would be disrupted, which could negatively affect shift workers’ physical and mental health (Wyse et al. 2017).

The circadian clock is a nearly 24-h endogenous timing mechanism that evolved in response to the Earth’s daily rotation and the resulting variations in light intensity. This clock, present in nearly all cell types, regulates key physiological functions, including hepatic processes, and its deregulation is linked to various chronic diseases, particularly liver disorders. Metabolic dysfunction-associated steatotic liver disease (MASLD), previously known as nonalcoholic fatty liver disease (NAFLD), was defined as hepatic steatosis accompanied by metabolic risk factors with no evidence of secondary causes of steatosis. The terminology shift was proposed in 2023 to better reflect the metabolic underpinnings of the disease (Rinella et al. 2023). Metabolic dysfunction-associated steatohepatitis (MASH) represents a more advanced stage within the MASLD spectrum, characterized by hepatic inflammation and cellular injury—paralleling the relationship between nonalcoholic steatohepatitis (NASH) and NAFLD. Without intervention, MASLD can progress to cirrhosis and hepatocellular carcinoma (HCC) (de Assis et al. 2023a, 2023b; Mukherji et al. 2019, 2020).

Several observational studies have investigated the association between shift work and MASLD. However, the findings have been inconsistent due to differences in study populations and methodologies. To better understand the connection between shift work and MASLD, we conducted this systematic review to summarize and evaluate the observational epidemiological evidence on this topic, which is crucial for developing effective interventions to mitigate the adverse effects of shift work on liver and metabolic health.

## Methods

### Design and protocol registration

The systematic review was conducted and reported according to recommendations from the Preferred Reporting Items for Systematic reviews and Meta-Analyses (PRISMA) statement (Page et al. 2021). The systematic review protocol was registered within the International Prospective Register of Systematic Reviews (PROSPERO) on 22 November 2024 (Registration number: CRD42024612749).

### Search strategy

We searched published articles using PubMed, Scopus, and Web of Science from the inception of each database to November 25, 2024. Exposure was identified using a range of terms related to shift and night work, while outcome terms included various descriptors of fatty liver disease, including MASLD, MASH, NAFLD, and NASH. Both MASLD and NAFLD were included as search terms, as evidence suggests that 99% of individuals diagnosed with NAFLD also meet the criteria for MASLD (Hagström et al. 2024). The hierarchical Medical Subject Headings (MeSH) terms “Non-alcoholic Fatty Liver Disease” and “Shift Work Schedule” in the PubMed database were used to optimize the inclusion of study exposure and outcome. The full search strategy is shown in Supplementary Table 1. Articles retrieved according to the search strategies were then imported to Covidence to remove duplicates, conduct independent screening, and resolve discrepancies.

Two independent authors scanned titles, abstracts, and full texts and included the studies that met the following inclusion criteria: (1) was a cohort, case–control, or cross-sectional study; (2) reported any type of shift work (e.g., regular night/morning work, 2-shift work, or other shift work schedule) as the exposure compared with the regular daytime work as the reference group, or any type of shift work in comparison with participants without that type of work; (3) reported MASLD as the study outcome. We excluded all animal studies, editorials, letters, comments, abstracts, posters, meta-analyses, and reviews. Studies reporting the impact of shift work on liver function without specifying MASLD as an outcome were also excluded.

### Data extraction and quality evaluation

We extracted information on the author, publication year, study design, sample size, follow-up time, participants’ age and gender, occupational group, measurements of shift work and MASLD, covariates, and study results. Both the crude and adjusted odds ratio (OR), relative risk (RR), incidence rate ratio (IRR), hazard ratio (HR), and 95% confidence interval (CI) were included as the risk estimates. The aforementioned data elements were extracted in a pre-designed Excel spreadsheet. The National Institutes of Health (NIH) quality assessment tool was used in evaluating the quality of included studies. The NIH quality assessment tool is a 14-item scale covering the clarity of the research question and study population, participation rate and application of inclusion and exclusion criteria, sample size justification, exposure and outcome measurement, sufficiency of timeframe, follow-up rate, and confounding adjustment (Ma et al. 2020; Stang 2010). Both data extraction and quality assessment were conducted by two authors independently. Agreement on key variables extraction was assessed, yielding a percent agreement of 94.4%, indicating high consistency. Interrater reliability for the quality assessment was measured using Cohen’s kappa, which was 0.59, indicating moderate agreement. All disagreements were resolved by consensus.

## Results

### Search results

**Fig. 1:**
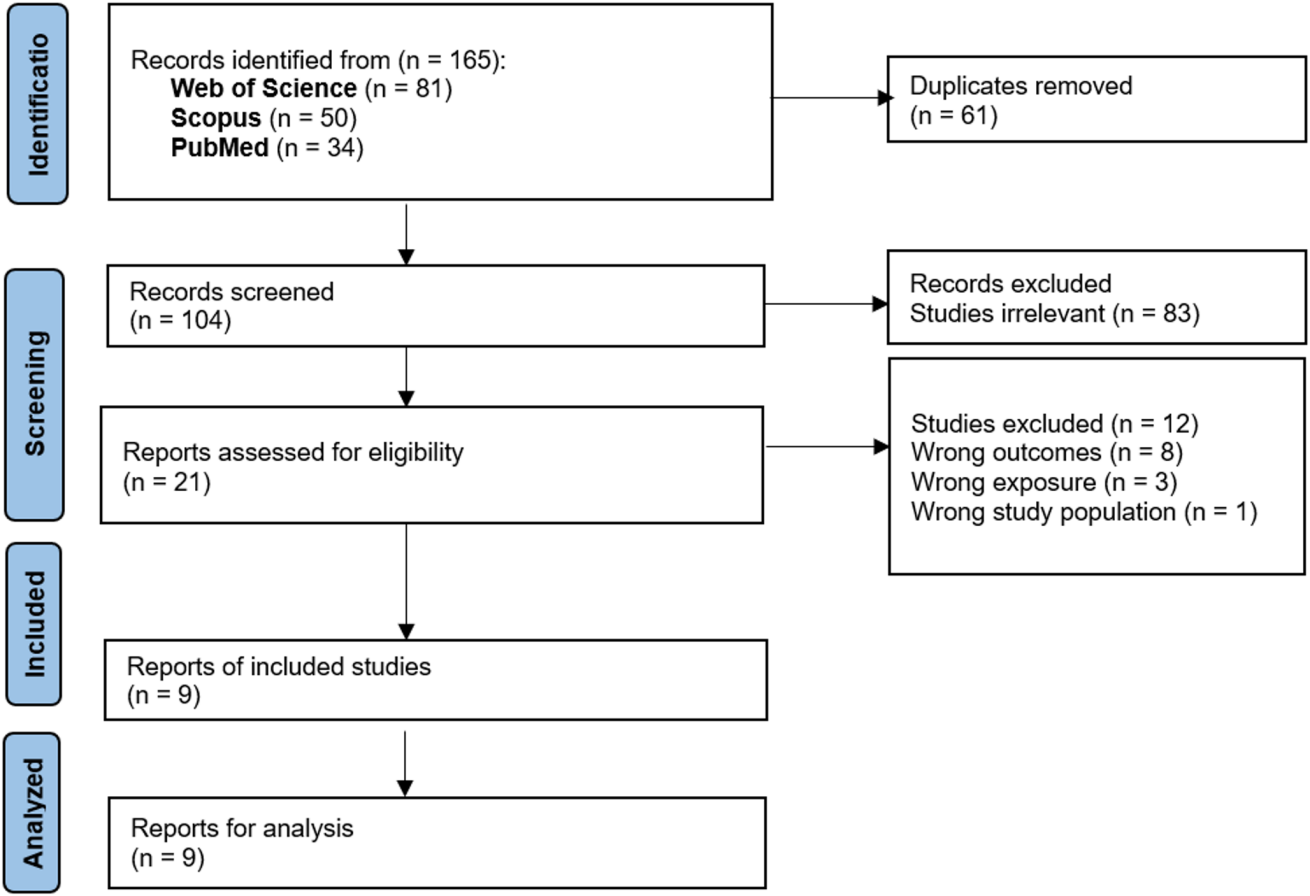
PRISMA flow chart including searches of databases

A total of 104 studies were retrieved through the search, of which 21 full-text studies were assessed for eligibility. Among them, 8 were excluded for wrong outcomes, 3 were excluded for wrong exposure, and one for wrong study population. Finally, nine articles were included in the systematic review (Balakrishnan et al. 2017; Che et al. 2024; Huang et al. 2023; Kim et al. 2022; Lee and Lee 2024; Maidstone et al. 2024; Taechasan and Jiamjarasrangsi 2024; Xu et al. 2023; Zhang et al. 2020). A flow chart for the review process is shown in Fig. 1.

### Characteristics of included studies

Study characteristics of included studies are summarized in Table 1 and the summary of key variables was displayed in Table 2. Among the nine included studies, six were conducted among the Asian population, one was from the U.S. based on the National Health and Nutrition Examination Survey (NHANES), and two other studies were based on the data from the UK Biobank. Regarding the study type, five of them were cross-sectional studies published from 2017 to 2024. Four cohort studies were published after 2022. The sample size ranged from 824 to 281,280, with a median of 8159. The occupational groups covered steel workers, railroad workers, healthcare professionals, academic personnel, security guards, and cleaning staff, while three studies did not specify the occupational types. The male-to-female ratio of the study participants varied by study and was closely related to the occupational type. Using the NIH quality assessment tool, studies were given an average score of 9.11 out of 14, ranging from 7 to 11. Common methodological issues include the absence of sample size justification, power description, or estimates of variance and effect, lack of repeated exposure measurement over time, and unblinded outcome measurement. The detailed scores for each item in each article are provided in Supplementary Table 2. Although MASLD is the updated terminology, all included studies used the previous term NAFLD, reflecting the diagnostic criteria in use at the time the studies were conducted. For consistency with the original studies, we retained the use of “NAFLD” when describing individual results.

**Table 1:** Characteristics of included studies

Author	Year	Country	Study design	Sample size	Follow-up time (year)	Ratio of male	Mean/median age (year)	Occupational group
Balakrishnan et al.	2017	United States	Cross-sectional	8,159	Not applicable	0.54	41.94	Not specified
Zhang et al.	2020	China	Cross-sectional	6,881	Not applicable	0.91	44.25	Steel workers
Kim et al.	2022	South Korea	Cross-sectional	2,511	Not applicable	All male workers	36.97	Steel workers
Xu et al.	2023	China	Cohort study	14,112	4	0.81	37.92	Railroad workers
Huang et al.	2023	UK	Cohort study	281,280	12.1	0.48	52.75	Not specified
Taechasan and Jiamjarasrangsi	2024	Thailand	Cohort study	3,620	7	0.18	40.97	Healthcare professionals, academic personnel, security guards, and cleaning staff
Maidstone et al.	2024	UK	Cross-sectional	276,043	Not applicable	0.47	53.16	Not specified
Lee and Lee	2024	South Korea	Cohort study	45,149	1–8	0.56	34.01	More than 80% of the screened participants were employees (and their spouses) of various business corporations and local governmental organizations who registered for health screening in their workplaces
Che et al.	2024	China	Cross-sectional	824	Not applicable	All female workers	32.62	Nurses

**Table 2:** Summary of key variables in included studies

Author	Measurement of exposure	Measurement of outcome	Covariates	Main study results	Subgroup and interaction analysis	Mediation effect
Balakrishnan et al.	Questionnaires	Liver enzyme levels	Age, race/ethnicity, sex, education, income, insurance, healthcare use, smoking, energy intake, BMI, WC, BP, HbA1c, TC, HDL, CRP, sleep hours	Shift work was not associated with NAFLD (OR 1.11, 95% CI 0.87–1.43)	Age, sex, race/ethnicity, BMI, WC, DM, CRP, HDL	Not available
Zhang et al.	Interviews verified with company records	Ultrasonography by blinded sonographers	Age, sex, BMI, smoking, drinking, DASH, education, physical activity, sleep duration, sleep quality, sedentary behavior, DM, DLP, BP, liver enzymes	Current night shift workers had higher odds of NAFLD (OR 1.23, 95% CI 1.02–1.48) vs. never night shift workers	Gender	Not available
Kim et al.	Questionnaires	Ultrasonography by blinded sonographers	Age, alcohol, smoking, physical activity, BMI, WC, employment duration, BP, glucose, lipidemia, liver function test, exposure to hepatotoxic materials	Shift workers had higher odds of moderate-severe NAFLD (OR 1.449, 95% CI 1.028–2.043) vs. daytime workers	Sleep disruption, nocturnal eating	Not available
Xu et al.	Questionnaires	Ultrasonography by blinded sonographers	Age, sex, BMI, smoking, diet, salt intake, physical activity, sleep, occupation, MAP, FBG, TC, TG, ALT, AST	RR of NAFLD was 1.069 (95% CI 0.998–1.146) and 1.179 (95% CI 1.059–1.312) for occasional and frequent shift workers, respectively, vs. seldom shift workers	Age, gender, BMI, smoking, diet, sleep duration, physical activity, salt intake, occupation	BMI and MAP
Huang et al.	Questionnaires	ICD-10 codes for hospital admissions or deaths	Age, sex, ethnicity, deprivation index, education, income, smoking, alcohol, physical activity, HTN, DM, PRS	Night shift work increased NAFLD risk: OR 1.12 (95% CI 0.96–1.31) for occasional and OR 1.27 (95% CI 1.08–1.48) for regular night shifts	Age, sex, deprivation index, education, income, smoking, physical activity, HTN, DM, PRS	BMI
Taechasan and Jiamjarasrangsi	Questionnaires	LAP and HSI	Sex, age, education, job type, marital status, HTN, DM, DLP, smoking, alcohol, exercise, BMI, WC, SBP, DBP, AST, ALT, FBG, TG, HDL	No association between current shift work and abnormal liver outcomes (e.g., ALT, AST, e-LAP, e-HSI)	Shift work duration, occupation type	Not available
Maidstone et al.	Questionnaires	DSI, ICD-10, and PDFF	Sex, age, ethnicity, deprivation index, sleep, alcohol, smoking, smoking pack-years, length of working week, BMI	Irregular shift workers had increased NAFLD risk (DSI OR 1.29, 95% CI 1.2–1.4)	Chronotype	BMI
Lee and Lee	Questionnaires	Ultrasonography by blinded sonographers	Age, sex, smoking, exercise, education, SBP, FBG, TC, BMI, HOMA-IR, PSQI	IRR for NAFLD in workers aged 20 s was 1.24 (95% CI 1.08–1.43)	Age, gender, sleep quality	Not available
Che et al.	Questionnaires	Abdominal B-ultrasound findings	Demographic characteristics, work situation, daily lifestyle, physical exam records	Monthly night shifts > 5 were independent predictors of NAFLD (OR 3.512, 95% CI 1.280–9.633)	Not available	Not available

*AIS* athens insomnia scale, *ALT* alanine aminotransferase, *AST* aspartate aminotransferase, *BMI* body mass index, *BP* blood pressure, *B-USG* B-mode ultrasonography, *CRP* C-reactive protein, *DASH* dietary approaches to stop hypertension, *DBP* diastolic blood pressure, *DLP* dyslipidemia, *DM* diabetes mellitus, *DSI* dallas steatosis index, *FBG* fasting blood glucose, *HbA1c* hemoglobin A1c, *HDL* high-density lipoprotein cholesterol, *HOMA-IR* homeostatic model assessment of insulin resistance, *HSI* hepatic steatosis index, *HTN* hypertension, *IRR* incidence rate ratio, *LAP* lipid accumulation product, *MAP* mean arterial pressure, *NAFLD* nonalcoholic fatty liver disease, *OR* odds ratio, *PDFF* proton density fat fraction, *PRS* polygenic risk score, *PSQI* Pittsburgh sleep quality index, *RR* relative risk, *SBP* systolic blood pressure, *TC* total cholesterol, *TG* triglycerides, *WC* waist circumference

### Measurement of exposure

Most studies collected shiftwork information through questionnaires. Zhang et al. (2020) collected the information through face-to-face personal interviews and further verified it with the company's records, which, to some degree, avoided the recall bias and increased the study’s validity.

Shift work exposure has been classified using various methods across studies, reflecting schedule, duration, and frequency differences. Three studies categorized the type of shift work based on whether it involved fixed/regular shifts or rotating/irregular shifts (Balakrishnan et al. 2017; Lee and Lee 2024; Maidstone et al. 2024). Taechasan and Jiamjarasrangsi (2024) classified participants into three groups: non-shift workers (those with no experience of night shift work or who worked fewer than four night shifts per month), former shift workers (those who previously worked more than four night shifts per month but were not engaged in shift work at the study's start), and current shift workers (those currently engaged in fixed or rotating night shift work for at least four nights per month). Several studies focused on metrics related to the duration and frequency of shift work. For example, Balakrishnan et al. (2017) classified shift workers by weekly hours worked (< 35, 36–40, or > 40 h). Che et al. (2024) and Taechasan and Jiamjarasrangsi (2024) categorized night shift frequency based on the number of night shifts per month. Additionally, Taechasan and Jiamjarasrangsi (2024), along with Kim et al. (2022), classified the total duration of shift work exposure by years, such as less than 10 years, 10–20 years, or more than 20 years. Xu et al. (2023) grouped participants based on the number of night shifts during the past year at baseline into seldom (< 1 time per week), occasional (1–3 times per week), and frequent (> 3 times per week) shift work categories. Huang et al. (2023) assessed exposure through multiple metrics, including duration (years of night shift work), frequency (average shifts per month), length (hours per shift), and consecutive shifts within a night shift period. Zhang et al. (2020) developed a comprehensive framework incorporating various metrics, including current/ever shift work, duration (years), cumulative number of night shifts (nights), cumulative length (hours), average frequency (nights/month), and average length (hours/night). The diverse classification systems used across studies underscore the complexity of measuring shift work exposure and highlight the need for standardized approaches to improve comparability and synthesis in future research.

### Measurement of outcome

Although no study utilized the gold standard liver biopsy for outcome assessment, various validated imaging and diagnostic methods were employed. Xu et al. (2023) and Lee and Lee (2024) utilized abdominal ultrasonography performed by experienced sonographers blinded to the study aims. Taechasan and Jiamjarasrangsi (2024) utilized diagnostic scores such as the lipid accumulation product (LAP) and hepatic steatosis index (HSI), both validated for sensitivity and specificity in prior research. Cross-sectional studies by Kim et al. (2022) and Zhang et al. (2020) also employed ultrasonography, interpreted by two blinded sonographers, to classify NAFLD severity into normal, light, and moderate-to-severe categories. Similarly, Che et al. (2024) relied on abdominal B-ultrasound findings as imaging evidence. In contrast, Balakrishnan et al. (2017) indirectly assessed NAFLD using liver enzyme levels due to the lack of imaging data in the NHANES cycles analyzed. Two studies based on the UK Biobank used different criteria. Huang et al. (2023) identified NAFLD using ICD-10 codes for hospital admissions or deaths, while Maidstone et al. (2024) adopted a more comprehensive approach, combining the Dallas steatosis index (DSI)—a logit model incorporating variables such as age, diabetes, hypertension, triglycerides, and ethnicity—with ICD-10 codes and hepatic proton density fat fraction (PDFF).

Several studies also implemented exclusion criteria to remove potential secondary causes. For example, Balakrishnan et al. (2017), Zhang et al. (2020), and Kim et al. (2022) excluded individuals with hepatitis B or C, as well as those with high alcohol consumption. Che et al. (2024) further excluded cases of drug-induced or autoimmune liver disease, while Lee and Lee (2024) excluded patients with cirrhosis based on ultrasonography and those taking medications for hepatitis, cirrhosis, or hepatic steatosis. To minimize alcohol-related confounding, Xu et al. (2023) excluded individuals with current or past excessive alcohol use and conducted sensitivity analyses on participants without liver diseases. Similarly, the two UK Biobank studies adjusted for alcohol intake frequency, with Huang et al. (2023) also performing a sensitivity analysis excluding participants with excessive alcohol consumption. Applying even stricter criteria, Taechasan and Jiamjarasrangsi (2024) excluded participants with abnormal liver function tests, such as elevated Aspartate Aminotransferase (AST), Alanine Aminotransferase (ALT), lipid accumulation product (LAP), or hepatic steatosis index (HSI), ensuring that only incident NAFLD cases were included in the analysis. These exclusion criteria help strengthen the focus on MASLD by minimizing the influence of other liver diseases or confounding factors.

Notably, temporality was not established in many of the included studies, particularly among those with cross-sectional designs. For example, Kim et al. (2022) and Zhang et al. (2020) assessed both shift work and NAFLD concurrently. Balakrishnan et al. (2017) also explicitly acknowledged that their study design precluded the assessment of a temporal sequence. Similarly, Maidstone et al. (2024) and Che et al. (2024) analyzed data from large cohort studies in a cross-sectional manner, offering no clear temporal direction. In contrast, cohort studies addressed temporality more effectively through their design. Huang et al. (2023) excluded participants with liver disease at baseline. Lee and Lee (2024) and Taechasan and Jiamjarasrangsi (2024) excluded individuals with a history of liver disease. Xu et al. (2023) excluded participants diagnosed with NAFLD prior to cohort entry, which demonstrated temporal alignment to support potential causal inferences between shift work and NAFLD.

### Covariates

Demographic factors were commonly adjusted for across studies, with several also considering socioeconomic variables, such as education level and household income (Balakrishnan et al. 2017; Zhang et al. 2020; Huang et al. 2023; Taechasan and Jiamjarasrangsi 2024; Maidstone et al. 2024; Lee and Lee 2024). Regarding lifestyle factors, studies consistently accounted for smoking status, alcohol consumption, and physical activity, with some incorporating more specific metrics, such as the Dietary Approaches to Stop Hypertension (DASH) score (Zhang et al. 2020) and sleep duration (Maidstone et al. 2024). Adjustments for clinical and metabolic factors varied among studies. For instance, body mass index (BMI) was controlled for in most of the studies (Balakrishnan et al. 2017; Zhang et al. 2020; Kim et al. 2022; Xu et al. 2023; Taechasan and Jiamjarasrangsi 2024; Maidstone et al. 2024; Lee and Lee 2024), while some studies also adjusted for liver function tests, including serum concentrations of liver enzymes (Kim et al. 2022; Xu et al. 2023). Additionally, work-related variables, such as job type and length of the working week, were adjusted for in studies by Taechasan and Jiamjarasrangsi (2024) and Maidstone et al. (2024).

### Study findings

Cross-sectional studies provided mixed results. For example, Kim et al. (2022) found that shift workers had higher odds of moderate-to-severe NAFLD compared to daytime workers (OR 1.499, 95% CI 1.028–2.043), although this association did not extend to light NAFLD. This result could be due to the limitations of abdominal ultrasound, which could not accurately distinguish between normal and light NAFLD. Zhang et al. (2020) identified night shift duration, cumulative number of night shifts, cumulative length of night shifts, and the frequency and length of night shifts as significant independent risk factors for NAFLD. Che et al. (2024) further reinforced the role of shift work intensity, identifying working more than five-night shifts per month as a strong independent risk factor for NAFLD prevalence (OR 3.512, 95% CI 1.280–9.633). Maidstone et al. (2024) provided different insights, showing that hepatic PDFF was elevated in irregular-shift workers but not in permanent night-shift workers, which implies that circadian misalignment may be a key factor driving pathological liver fat accumulation in certain groups of shift workers. In contrast, Balakrishnan et al. (2017) reported no significant association between shift work and NAFLD risk (OR 1.11, 95% 0.87–1.43). The reliance on enzyme-based assessments rather than imaging for diagnosis may have contributed to this null finding, as enzyme-based measures might lack the sensitivity required to detect early or subclinical NAFLD.

Regarding the longitudinal analyses, Huang et al. (2023) identified a dose–response relationship between night shift work and NAFLD risk. Workers engaging in occasional night shifts (OR 1.12, 95% CI 0.96–1.31) or permanent night shifts (OR 1.27, 95% CI 1.08–1.48) were at higher risk of developing NAFLD compared to those who never or rarely worked night shifts. Moreover, among 75,059 participants with lifetime shift work data, longer duration, higher frequency, more consecutive night shifts, and longer shift lengths were consistently associated with elevated NAFLD risk. Similarly, Xu et al. (2023) also found that frequent shift work, but not occasional shift work, was a significant risk factor for NAFLD in rail workers (HR = 1.179, 95% CI 1.059–1.312).

### Subgroup and interaction analysis

A growing body of research has explored how demographic, lifestyle, chronotype, genetic, and occupational factors modify the relationship between shift work and MASLD risk. For example, Lee and Lee (2024) suggested shift work significantly increased NAFLD risk in women (IRR = 1.39, 95% CI 1.05–1.84) in their 20s but not in other gender and age groups, suggesting that younger women may be more susceptible to the adverse effects of shift work. Conversely, Zhang et al. (2020) reported a significant association between night shift work and NAFLD among male workers but not among females, likely due to the small number of female workers and the low prevalence of NAFLD in this group, which may have limited the accuracy of interval estimates.

Lifestyle factors also play a crucial role. Balakrishnan et al. (2017) found a significant association between shift work and NAFLD only in individuals with normal BMI. Kim et al. (2022) observed that workers prohibited from sleeping during shifts and those who consumed food at night had a higher risk of moderate-to-severe NAFLD. Similarly, Lee and Lee (2024) found that shift work was significantly associated with an increased risk of NAFLD, especially among individuals with poor sleep quality and those in their 20s (IRR = 1.37, 95% CI 1.11–1.70). Xu et al. (2023) found that the NAFLD risk associated with occasional shift work was higher among individuals who had never smoked and maintained a balanced diet, and the NAFLD risk associated with frequent shift work was elevated among those with never smoking, a balanced dietary pattern, middle salt intake, seldom physical activity, and moderate sleep duration, although the interaction was not statistically significant. These findings highlight the complex relationships between shift work and lifestyle factors.

Two studies explored the potential effect modification by chronotype and genetic factors. Maidstone et al. (2024) examined chronotype and found that patients with extreme late chronotypes had a higher likelihood of NAFLD (OR 1.45, 95% CI 1.34–1.56 for DSI-defined NAFLD; OR 1.23, 95% CI 1.09–1.39 for ICD-10-diagnosed NAFLD). In addition, the association between shift work and DSI was stronger among morning types than among evening types (P-interaction < 0.01). Huang et al. (2023) used polygenic risk scores (PRS) to assess genetic susceptibility to NAFLD in individuals of European descent. Despite variations in genetic risk, the study found no significant interaction between genetic predisposition and the relationship between night shift work and incident NAFLD (P-interaction: 0.151–0.386), indicating that genetic risk does not significantly modify the effects of shift work on liver health.

Findings from Taechasan and Jiamjarasrangsi (2024) also reveal a relationship between shift work duration, occupation type, and NAFLD risk. Healthcare workers with 10–20 years of shift work had a higher risk of elevated lipid accumulation product (e-LAP), whereas those with over 20 years of exposure had a lower risk. In contrast, non-healthcare workers with less than 10 years of shift work had a higher risk of elevated hepatic steatosis index (e-HSI). These findings suggest that prolonged shift work may lead to adaptive changes in healthcare workers, potentially reducing NAFLD risk. The decreasing workload of shift workers in Thailand as shift work duration increases, particularly healthcare personnel, may also help explain these results.

### Mediation effect of obesity and BMI

The role of BMI and obesity in mediating the relationship between shift work and MASLD has been demonstrated in several studies. Huang et al. (2023) conducted a mediation analysis, revealing that BMI accounted for 31.7% (95% CI 14.3–56.4, *P* < 0.001) of the association between current night shift work and NAFLD development. Xu et al. (2023) also examined the mediating role of metabolic factors, finding that higher BMI, mean arterial pressure (MAP), fasting blood glucose (FBG), total cholesterol (TC), triglycerides (TG), ALT, and AST directly contributed to NAFLD occurrence, while the indirect effects of FBG, TC, TG, ALT, and AST were not statistically significant. Maidstone et al. (2024) obtained similar results that approximately 36% of the relationship between shift work and NAFLD was mediated by BMI (*P* < 0.01), underscoring the mediating role of BMI in this pathway.

## Discussion

This systematic review aimed to synthesize the evidence on the association between shift work and MASLD. In general, studies consistently show that shift work is a risk factor for MASLD with a dose-dependent relation. The positive association is also consistent with published results regarding abnormal liver function and rotating shift work (Lin and Chen 2015; Wang et al. 2019). On the other hand, some studies, such as Balakrishnan et al., failed to detect a significant link between shift work and liver disease, potentially due to differences in measurement. Subgroup and interaction analyses within these studies further suggested that gender, age, lifestyle, chronotype, and occupational factors may modify the impact of shift work on MASLD risk. Given the growing prevalence of shift work and its potential health consequences, these findings underscore the need for targeted public health interventions and occupational policies to mitigate the risk of MASLD among shift workers. Optimizing shift schedules is crucial for reducing the adverse effects associated with shift work—for example, by limiting consecutive night shifts and ensuring adequate rest periods between shifts (Wickwire et al. 2017). Additionally, promoting healthy lifestyles may further reduce the risk of MASLD, while regular health assessments can support the early detection and management of metabolic abnormalities.

The biological association between shift work and MASLD has also been reported in previous studies, with mechanisms involving several pathways. Shift work is associated with reduced melatonin secretion due to exposure to artificial light at night. Melatonin has antioxidant and anti-inflammatory properties that protect hepatocytes from oxidative stress (Wei et al. 2008). Therefore, melatonin suppression in shift workers leads to increased reactive oxygen species (ROS) production, accelerating hepatic lipid peroxidation and promoting MASLD progression (Ahammed et al. 2024; Wei et al. 2020). Insulin resistance is also a well-documented consequence of circadian disruption. Studies have shown that shift workers exhibit higher fasting glucose levels, increased insulin resistance, and impaired glucose tolerance, all of which are key contributors to MASLD pathogenesis (Strohmaier et al. 2018; Tan and Scott 2014). Moreover, leptin resistance is common in shift workers, leading to increased food intake, weight gain, and adiposity, further exacerbating the risk of MASLD (Ji et al. 2019). Additionally, shift work alters glucocorticoid secretion patterns, particularly cortisol, which plays a role in hepatic gluconeogenesis and lipid metabolism. Chronically elevated cortisol levels due to stress and circadian misalignment promote visceral fat deposition and hepatic lipid accumulation (Tsai et al. 2022; Woods et al. 2015).

Additionally, potential mechanisms reported by previous studies may explain the stronger effects of shift work observed in certain subgroups, such as young individuals, females, and those who eat during shift work. For instance, research has shown that younger individuals are more vulnerable to sleep restriction than older adults (Molzof et al. 2022). The healthy worker effect, which suggests that healthier individuals are more likely to tolerate shift work as they age, should also be considered. Moreover, hormonal responses to sleep deprivation differ by sex, and women experience a more significant decrease in serum leptin levels following sleep deprivation compared to men (van Egmond et al. 2023). Eating patterns during shift work have also been implicated in adverse metabolic outcomes. Studies have shown that consuming most calories at night (21:00–06:00) is associated with significantly higher insulin and leptin levels compared to daytime eating, which may exacerbate metabolic dysfunction (Molzof et al. 2022).

Notably, a number of methodological limitations were prevalent among the included studies, which may affect the accuracy and interpretation of the findings. First, among all included studies, only one verified the self-reported exposure information by work records, which may limit accuracy and reliability due to recall bias. In addition, not all studies were able to capture data on the lifetime duration of shift work. Second, some studies did not specify the strength or type of shift work due to the limited data collection, which may lead to workload misclassification and bias the association between shift work and liver diseases towards the null. The lack of standardized measures for shift work also contributes to inconsistencies in observed associations, making it challenging to compare findings across studies. Third, only four studies mentioned the use of blindness in outcome measurement, which may reduce the accuracy of outcome measurement. Fourth, none of the studies incorporated time-varying analysis, which overlooks the dynamic changes in working patterns and covariates, potentially leading to biased risk estimates. Fifth, although a few studies explored the mediating effect of obesity and BMI, the impact of other relevant health behaviors, such as social jet lag, irregular mealtimes, poor diet, and work-related environmental factors, also warrants consideration. The scope of the studies included also remains limited. Most studies focused on manual workers or did not specify occupational groups, with only two studies reporting results for healthcare professionals. Additionally, the majority of studies were conducted among White and Asian populations, providing limited data on other demographic groups. These limitations highlight significant knowledge gaps that future research should address.

This systematic review has several limitations. First, we were unable to conduct a meta-analysis on shift work and MASLD due to heterogeneity in population characteristics, work environments, covariate selection, and exposure and outcome definitions. Second, we only included observational studies, which may introduce inherent biases such as confounding and selection bias. Among them, only four cohort studies explicitly excluded participants with MASLD at baseline, thereby limiting the temporality and ability to establish causal relationships. Therefore, the overall evidence should be interpreted with caution. Addressing these limitations in future research could help clarify the relationship between shift work and MASLD and inform the development of effective interventions.

## Conclusion

In conclusion, while a generally consistent association between shift work and MASLD exists, establishing a clear link between shift work and MASLD is challenging due to methodological issues in study designs. In addition, the magnitude of this association varies by factors such as gender, age, lifestyle, chronotype, and occupational types. More rigorous prospective studies are needed, incorporating comprehensive and dynamic data on exposure, outcomes, and covariates, as well as a diverse participant population. Additional animal and cell studies are also warranted to further understand the biological basis and causal relationship between shift work and MASLD. Targeted public health interventions and occupational policies are required to mitigate the risk of MASLD among shift workers.

## Electronic Supplementary Material

Below is the link to the electronic supplementary material.

Supplementary Material

## Data Availability

This study is a systematic review of published literature. All data analyzed during this review are included in the published articles cited in the manuscript. No new datasets were generated or analyzed by the authors. The full search strategy and quality assessment details are available in supplementary materials.
