# Supplementary material for: Shift work and metabolic dysfunction-associated steatotic liver disease: a systematic review of observational studies
Source: Int Arch Occup Environ Health. 2025 Sep 17;98(9-10):777–86. doi: 10.1007/s00420-025-02171-6 (PMC12672679; doi:10.1007/s00420-025-02171-6)
Supplement: Supplementary file 1 — Supplementary Material [file 420_2025_2171_MOESM1_ESM.docx]

Article title: Shift Work and Metabolic Dysfunction-Associated Steatotic Liver Disease: A Systematic Review of Observational Studies

Journal name: International Archives of Occupational and Environmental Health

Bingya Ma^1*^, Yihang Fan^1^, Wenjun Fan^1^

1. Department of Epidemiology and Biostatistics, University of California, Irvine, Irvine, CA, United States of America

* Corresponding author

Email: [bingyam@uci.edu](mailto:bingyam@uci.edu)

**Supplementary Table 1** Search Strategy

| **Database** | **Search Strategy** |
| --- | --- |
| Web of Science | TS=("fatty liver" OR steatohepatit* OR steatos* OR NAFLD OR NASH OR "Non-alcoholic Fatty Liver Disease" OR steatotic OR MASLD OR MAFLD OR MASH)  AND  TS=("shift work" OR "night work" OR shift-work OR shiftwork OR "shift worker" OR "shift-worker" OR "shift-workers" OR shiftworker OR "shift workers" OR "irregular hours" OR "rotating shift") |
| Scopus | (TITLE-ABS-KEY("fatty liver" OR steatohepatit* OR steatos* OR NAFLD OR NASH OR "Non-alcoholic Fatty Liver Disease" OR steatotic OR MASLD OR MAFLD OR MASH)  AND  TITLE-ABS-KEY("shift work" OR "night work" OR shift-work OR shiftwork OR "shift worker" OR "shift-worker" OR "shift-workers" OR shiftworker OR "shift workers" OR "irregular hours" OR "rotating shift")) |
| PubMed | ("fatty liver" OR steatohepatit* OR steatos* OR NAFLD OR NASH OR steatotic OR MASLD OR MAFLD OR MASH OR "Non-alcoholic Fatty Liver Disease"[Mesh])  AND  ("shift work" OR "night work" OR shift-work OR shiftwork OR "shift worker" OR "shift-worker" OR "shift-workers" OR shiftworker OR "shift workers" OR "shift work" OR shiftwork OR "irregular hours" OR "rotating shift" OR "Shift Work Schedule"[Mesh]) |

**Supplementary Table 2** NIH Quality Assessment Tool for Observational Cohort and Cross-Sectional Studies

| **Criteria** | **Balakrishnan et al. 2017** | **Zhang et al. 2020** | **Kim et al. 2022** | **Xu et al. 2023** | **Huang et al. 2023** | **Taechasan & Jiamjarasrangsi 2024** | **Maidstone et al. 2024** | **Lee & Lee 2024** | **Che et al. 2024** |
| --- | --- | --- | --- | --- | --- | --- | --- | --- | --- |
|  |  |  |  |  |  |  |  |  |  |
| 1. Was the research question or objective in this paper clearly stated? | Yes | Yes | Yes | Yes | Yes | Yes | Yes | Yes | Yes |
| 2. Was the study population clearly specified and defined? | Yes | Yes | Yes | Yes | Yes | Yes | Yes | Yes | Yes |
| 3. Was the participation rate of eligible persons at least 50%? | Yes | Yes | No | No | Yes | No | Yes | No | No |
| 4. Were all the subjects selected or recruited from the same or similar populations (including the same time period)? Were inclusion and exclusion criteria for being in the study prespecified and applied uniformly to all participants? | Yes | Yes | Yes | Yes | Yes | Yes | Yes | Yes | Yes |
| 5. Was a sample size justification, power description, or variance and effect estimates provided? | No | No | No | No | No | No | No | No | No |
| 6. For the analyses in this paper, were the exposure(s) of interest measured prior to the outcome(s) being measured? | No | No | No | Yes | Yes | Yes | No | Yes | Yes |
| 7. Was the timeframe sufficient so that one could reasonably expect to see an association between exposure and outcome if it existed? | No | No | No | Yes | Yes | Yes | No | Yes | No |
| 8. For exposures that can vary in amount or level, did the study examine different levels of the exposure as related to the outcome (e.g., categories of exposure, or exposure measured as continuous variable)? | Yes | Yes | Yes | Yes | Yes | Yes | Yes | No | No |
| 9. Were the exposure measures (independent variables) clearly defined, valid, reliable, and implemented consistently across all study participants? | Yes | Yes | Yes | Yes | Yes | Yes | Yes | Yes | Yes |
| 10. Was the exposure(s) assessed more than once over time? | No | No | No | No | No | No | No | No | No |
| 11. Were the outcome measures (dependent variables) clearly defined, valid, reliable, and implemented consistently across all study participants? | Yes | Yes | Yes | Yes | Yes | Yes | Yes | Yes | Yes |
| 12. Were the outcome assessors blinded to the exposure status of participants? | No | Yes | Yes | Yes | No | No | No | Yes | No |
| 13. Was loss to follow-up after baseline 20% or less? | No | No | No | Yes | Yes | Yes | No | Yes | No |
| 14. Were key potential confounding variables measured and adjusted statistically for their impact on the relationship between exposure(s) and outcome(s)? | Yes | Yes | Yes | Yes | Yes | Yes | Yes | Yes | Yes |
| Total | 8 | 9 | 8 | 11 | 11 | 10 | 8 | 10 | 7 |
